# Advancing the Compositional Analysis of Olefin Polymerization Catalysts with High-Throughput Fluorescence Microscopy

**DOI:** 10.1021/jacs.2c09159

**Published:** 2022-11-08

**Authors:** Maximilian J. Werny, Kirsten B. Siebers, Nicolaas H. Friederichs, Coen Hendriksen, Florian Meirer, Bert M. Weckhuysen

**Affiliations:** † Inorganic Chemistry and Catalysis Group, Institute for Sustainable and Circular Chemistry and Debye Institute for Nanomaterials Science, Utrecht University, Universiteitsweg 99, 3584CG Utrecht, The Netherlands; ‡ Dutch Polymer Institute (DPI), P.O. Box 902, 5600AX Eindhoven, The Netherlands; § SABIC Technology Center, Urmonderbaan 22, 6167RD Geleen, The Netherlands

## Abstract

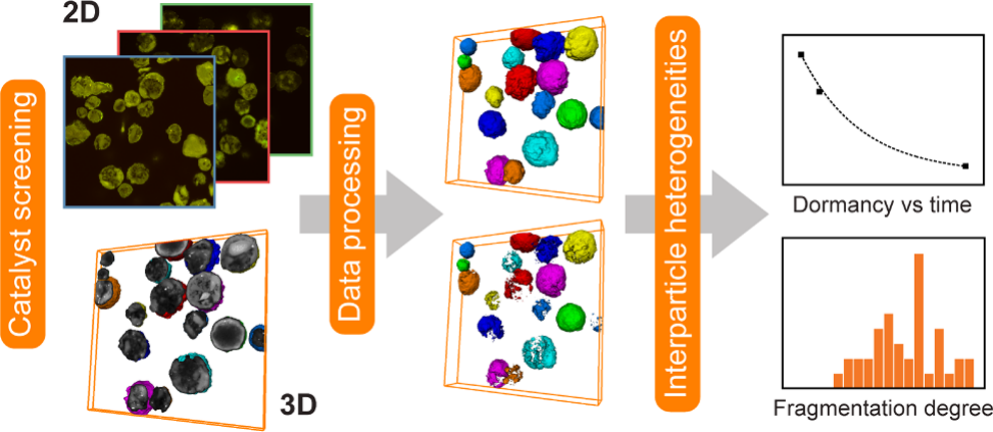

To optimize the performance of supported olefin polymerization catalysts, novel methodologies are required to evaluate the composition, structure, and morphology of both pristine and prepolymerized samples in a resource-efficient, high-throughput manner. Here, we report on a unique combination of laboratory-based confocal fluorescence microscopy and advanced image processing that allowed us to quantitatively assess support fragmentation in a large number of autofluorescent metallocene-based catalyst particles. Using this approach, significant inter- and intraparticle heterogeneities were detected and quantified in a representative number of prepolymerized catalyst particles (2D: ≥135, 3D: 40). The heterogeneity that was observed over several stages of slurry-phase ethylene polymerization (10 bar) is primarily attributed to the catalyst particles’ diverse support structures and to the inhomogeneities in the metallocene distribution. From a mechanistic point of view, the 2D and 3D analyses revealed extensive contributions from a layer-by-layer fragmentation mechanism in synergy with a less pronounced sectioning mechanism. A significant number of catalyst particles were also found to display limited support fragmentation at the onset of the reaction (i.e., at lower polymer yields). This delay in activity or “dormancy” is believed to contribute to a broadening of the particle size distribution during the early stages of polymerization. 2D and 3D catalyst screening via confocal fluorescence microscopy represents an accessible and fast approach to characterize the structure of heterogeneous catalysts and assess the distribution of their fluorescent components and reaction products. The automation of both image segmentation and postprocessing with machine learning can yield a powerful diagnostic tool for future research as well as quality control on industrial catalysts.

## Introduction

Silica-supported metallocenes represent a promising class of industrial olefin polymerization catalysts due to their high activities and their ability to produce polyolefins with tailored properties.^1,2^ The single-site character of their active sites essentially facilitates the production of narrow molecular weight polymers with well-defined tacticity and co-monomer incorporation.^3,4^ Industrial supported metallocene-based catalysts typically consist of highly porous amorphous silica particles in a size range of 20–100 μm, impregnated with a group 4 transition-metal complex, usually zirconium-based, and methylaluminoxane (MAO) as a co-catalyst.^5^ The immobilization of the metallocene limits reactor fouling and, moreover, ensures a uniform morphology and high bulk density of the produced polymer particles.^6^

Both the activity and morphological evolution of the supported catalysts are governed by the phenomenon of fragmentation, that is, the disintegration of the catalyst support due to polymer formation.^7,8^ The process releases new active sites and promotes homogeneous particle growth (replica effect), thereby limiting the formation of fines, which also contributes to reactor fouling.^9−11^ Fragmentation plays an important role in overcoming mass- and heat-transfer limitations, which would otherwise severely affect the catalyst performance and product properties.^6,8^ Thus, to optimize the existing catalyst designs as well as to improve the physical and mechanical properties of the formed polymers, a more comprehensive understanding of the factors controlling the process of support fragmentation is necessary.

A common approach to evaluate the internal morphology of supported polymerization catalyst particles involves accessing particle cross sections via microtoming or focused ion beam (FIB) cutting and subsequent imaging via scanning electron microscopy (SEM).^12−19^ Despite yielding highly resolved morphological data, this approach remains laborious, destructive and, moreover, does not provide 3D resolved data. Synchrotron- and laboratory-based X-ray nanotomography experiments, on the other hand, provide unparalleled 3D imaging capabilities at high spatial resolutions but are elaborate in terms of sample preparation, experimental execution, and data analysis.^20−23^ Both approaches, moreover, deliver limited physicochemical and catalytic information due to their low sample throughput. While multiple olefin polymerization catalyst particles have recently been studied with hard X-ray nanotomography,^21^ the characterization of a large number of particles was facilitated by the comparatively small average particle size of the investigated catalyst (i.e., 5.9 μm).

**Figure 1:**
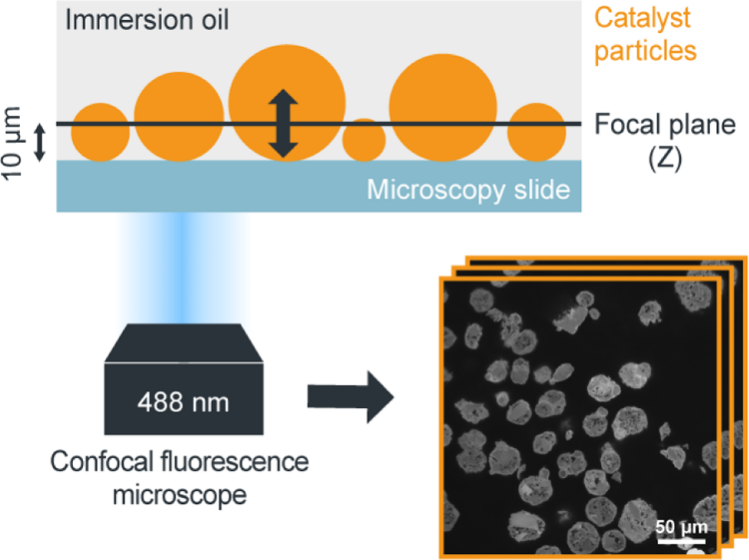
Schematic illustration of the high-throughput CFM approach that was employed for the characterization of the autofluorescent Zr/MAO/SiO_2_ catalyst samples. Multiple metallocene-based catalyst particles were excited with a 488 nm laser and scanned at different focal depths using an oil immersion objective to obtain *Z*-stacks of fluorescence microscopy images. These *Z*-stacks were then converted into 3D tomographies by means of image processing. To record and compare 2D data, all samples were measured at a fixed focal depth of 10 μm.

In this work, we present a more accessible approach for multiparticle analysis based on confocal fluorescence microscopy (CFM). The laboratory-based technique can deliver both 2D and 3D morphological data at a high sample throughput due to its large field of view (FOV) and short measurement times (2D: <1 min for 178 μm × 178 μm FOV, 3D: ∼2 h for 178 μm × 178 μm × 30 μm) (Figure 1). Fluorescence microscopy is widely used in the field of life sciences to selectively visualize cellular components and processes, usually in combination with fluorescent probe molecules.^24−27^ Its application in the field of catalysis is, however, more recent and ranges from the investigation of, among others, the pore space architecture in catalyst extrudates to mapping Brønsted acidity in industrial catalysts.^28−35^ Specifically, in the context of olefin polymerization catalysis, fluorescence microscopy has been employed to visualize monomer incorporation and the formation of nascent polymers,^36−39^ as well as to qualitatively assess support fragmentation in individual catalyst particles.^40−43^ Building on this, we employed fluorescence microscopy in combination with advanced image processing to obtain quantitative insights into the morphology of a large number of silica-supported zirconocene-based catalyst particles that display autofluorescence.

As CFM does not require intensive sample preparation and, moreover, facilitates high sample throughput, it represents an attractive laboratory-based alternative to X-ray-based experimentation for assessing the morphology of extensive sample sets. Ultimately, it can be used as a high-throughput tool to assess the quality and state of different heterogeneous catalysts after synthesis, as well as after reaction. Taking the autofluorescent zirconocene-based catalyst as an example, the spatial distribution of the metallocene on the support delivers information on the quality of the pristine catalyst after synthesis. Ideally, the support is homogeneously impregnated with the metallocene. In prepolymerized particles, the fluorescence of the metallocene directly yields the distribution of the catalyst support. This, retrospectively, delivers information on the catalyst’s morphological behavior during polymerization. Similar insights may be gained for other heterogeneous catalysts, either via staining approaches with chemosensitive- and/or size-selective probes or by forming fluorescent reaction products (e.g., coke, thiophene, or styrene oligomerization products and fluorophore-tagged polymers), thus enabling a selective visualization of specific catalyst domains, pore space architectures, and catalytic reactions.^34,38,44−48^ Automating the data acquisition and analysis with machine learning could ultimately yield statistically relevant insights into the behavior of heterogeneous catalysts and, possibly, enable us to derive quantitative structure–activity correlations.

## Results and Discussion

### Catalyst Synthesis and Prepolymerization

In this study, we have investigated a silica-supported bis-indenyl zirconocene-based catalyst (Zr/MAO/SiO_2_) that was previously studied by our group during gas-phase ethylene polymerization.^19^ The catalyst was synthesized by suspending a 2,2′-biphenylene-bis-2-indenyl ZrCl_2_ complex and MAO(Al/M ratio = 150) in dried toluene, subsequently adding polymer-grade SiO_2_ (*D*_50_ = 25.0 μm; precalcination at 600 °C) to form a slurry, and removing the solvent under N_2_ flow. Further details can be found in Section S1 of the Supporting Information (SI).

**Figure 2:**
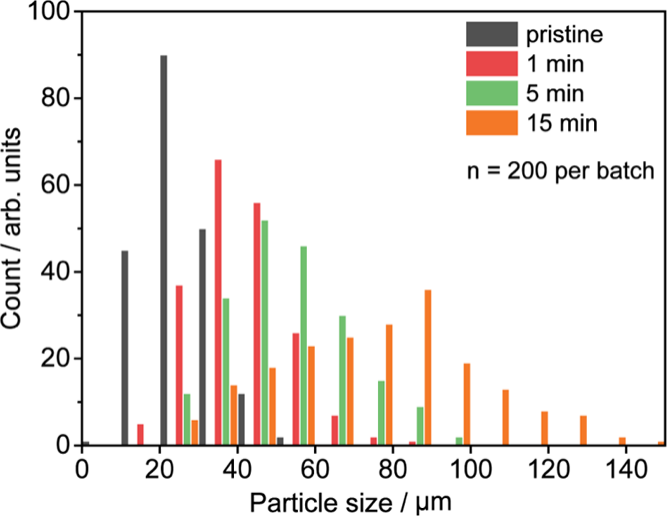
PSD of the pristine (black), 1 min (red), 5 min (green), and 15 min (orange) prepolymerized Zr/MAO/SiO_2_ catalyst samples (slurry phase, 10 bar ethylene, and room temperature). A total of 200 catalyst particles were assessed per batch.

The catalyst was then prepolymerized in dried heptane in an autoclave at 10 bar ethylene pressure for 0.5, 1, 5, and 15 min, respectively (room temperature; Supporting Information, Section S2). As can be seen in Table 1 and Figure 2, the average particle size increases with the reaction time. Furthermore, a concurrent broadening of the particle size distribution (PSD), as indicated by an increasing standard deviation (SD), points to kinetic differences among the individual particles of the prepolymerized batches. This stands in agreement with other works, where optical microscopy revealed kinetic differences among the catalyst particles.^49−55^

**Table 1:** Yields, *D*_50_ Values, and Average Particle Sizes of the Pristine and Selected 10 bar Prepolymerized Zr/MAO/SiO_2_ Catalyst Samples, as Determined via Optical Microscopy for 200 Catalyst Particles^a^

sample	yield/g_PE_/g_cat_	*D*_50_/μm	*d*_avg_/μm	SD/%
pristine	0	25.8	26.7	8.4
1 min	2.1	38.4	39.8	11.7
5 min	4.8	51.1	52.1	15.2
15 min	18.5	74.2	74.5	25.7

a *d*_avg_ = average particle diameter; SD = standard deviation.

### Screening the Fragmentation Degree and Catalyst Particle Dormancy over Multiple Reaction Stages with 2D CFM

**Figure 3:**
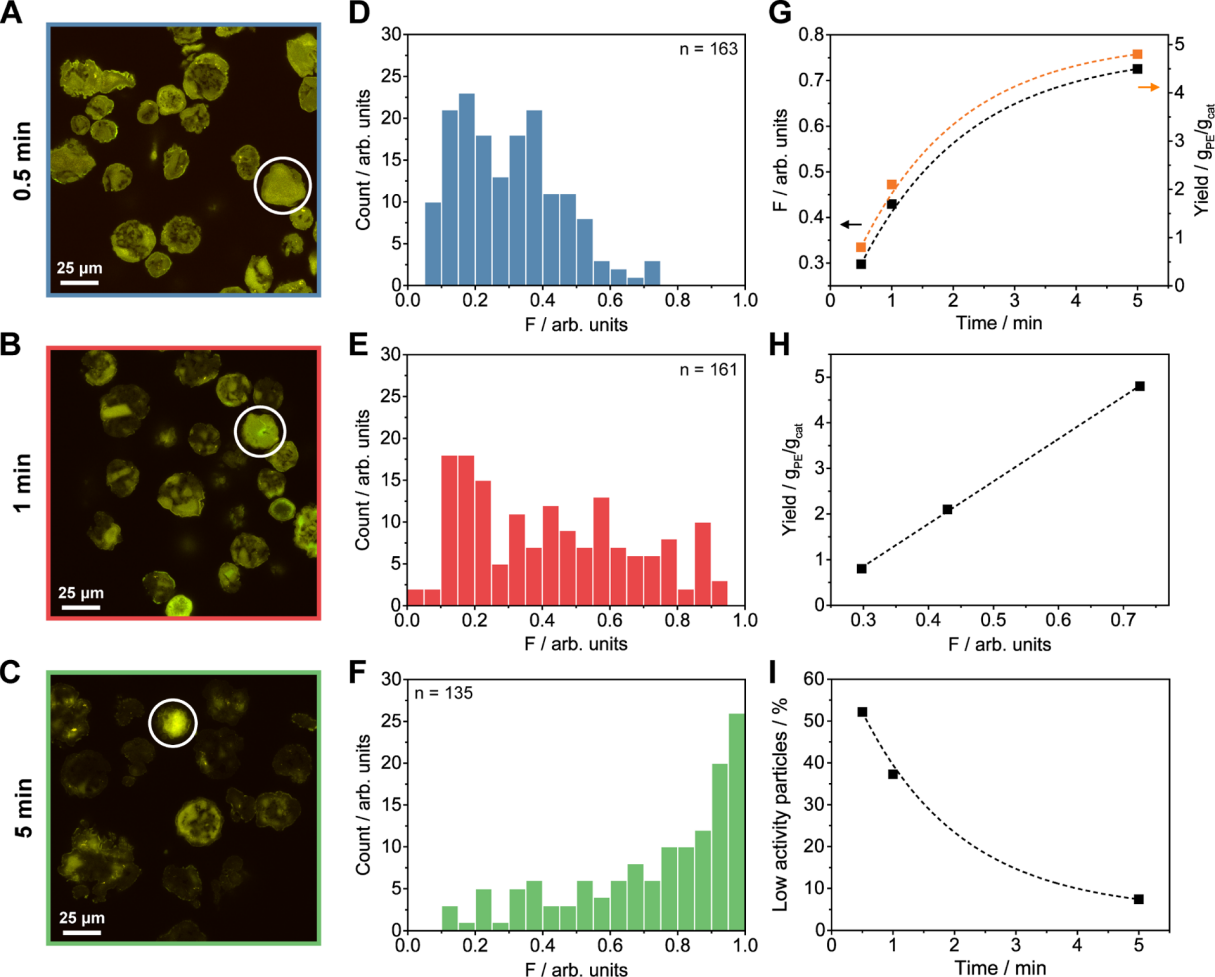
Two-dimensional CFM data acquired of the Zr/MAO/SiO_2_ catalyst at different reaction stages (0.5, 1, 5 min; 10 bar ethylene; room temperature; 0.8–4.8 g_PE_/g_cat_). (A–C) 2D CFM images of the characterized particles. (D–F) Histogram of the particles’ respective fragmentation parameters with *F* = *A*_P_/TPA; (G) average *F* and PE yield per reaction stage plotted vs the prepolymerization time; (H) average *F* per reaction stage plotted vs the corresponding PE yield; and (I) percentage of particles, with the value of *F* smaller than the average *F* value of the 0.5 min prepolymerized batch (*F* < 0.30), indicating a lower degree of polymerization and thus lower relative activity (i.e., dormancy). For each reaction stage, a dormant catalyst particle has been marked with a circle (A–C).

Two-dimensional CFM was used to assess the morphology and fragmentation of the Zr/MAO/SiO_2_ catalyst, both qualitatively and quantitatively, at multiple reaction stages (Table 2 and Figure 3; Supporting Information, Sections S3 and S4; samples exposed to air). In contrast to previous CFM studies performed on similar systems,^40,41,56^ no chemical modification of the catalyst, that is, via impregnation with suitable fluorophores, had to be performed due to the autofluorescent nature of the metallocene-based Zr/MAO/SiO_2_ catalyst (Figure S1; both SiO_2_ and MAO/SiO_2_ are nonfluorescent, Figure S2). The CFM data (Figures 3A–C; Supporting Information, Figures S3–S7) were interpreted based on the cross-sectional analysis of randomly selected prepolymerized catalyst particles with FIB–SEM (Figures S8–S11): high fluorescence intensity regions represent support-dominant domains (pure silica + silica-dominant mixed phase, denoted as *A*_S_), while low fluorescence intensity regions are predominantly constituted by polyethylene (PE), PE-dominant mixed phase, and the macropore space (in sum denoted as *A*_P_). In general, a decrease in fluorescence intensity was observed in areas where the support is diluted with the formed polymer. All images for analysis were acquired using a large FOV (178 μm × 178 μm) at a fixed focal depth of ∼10 μm. The latter helped to obtain fluorescence intensities that are still sufficiently high for reliable characterization and image processing, while also ensuring comparability of the data. A lateral resolution of ∼470 nm was determined via line scan analysis of the 2D images of the pristine catalyst (Figure S12).

**Table 2:** Quantitative Data Extracted via 2D CFM and Image Processing for the 0.5 min, 1 min, and 5 min Prepolymerized Zr/MAO/SiO_2_ Catalyst Batches (10 bar Ethylene)^a^

sample	yield/g_PE_/g_cat_	*n*	*F*_avg_	SD/%	*d*_Feret__,avg_/μm	PCC
0.5 min	0.8	163	0.30	15.2	26.6	–0.20
1 min	2.1	161	0.43	24.7	29.0	–0.14
5 min	4.8	135	0.73	24.7	35.8	–0.15

a *n* = total number of full or partial particle cross sections; *F*_avg_ = average fragmentation parameter *F* of all particles belonging to a sample; SD = standard deviation of *F*; *d*_Feret, avg_ = average 2D Feret diameter of a sample; and PCC = Pearson’s correlation coefficient for *F* and *d*_Feret_.

The 2D CFM images of the three investigated polymerization stages (Figures 3A–C and S4–S6) show that both the polymerization degree (i.e., the amount of formed polymer and the degree of internal support fragmentation) and average particle size increased with reaction progress (Table 2). From a qualitative point of view, a large degree of inter- and intraparticle heterogeneities is clearly evident. In most particles, the layer-by-layer fragmentation mechanism dominates at both particle and silica domain levels (Figures S4–S7). This is evident from a gradual change in fluorescence intensity at the perimeter of the catalyst particles’ constituent support granulates, indicating progressing polymerization and support fragmentation (see the differences in fluorescence intensity between pristine and prepolymerized catalyst particles in Figures S3 and S4 for clarification; we also refer here to the SEM images in Figure S8). The sectioning mechanism, on the other hand, is less prominent. In fact, it is mostly involved in cleaving larger, inaccessible support fragments with low degrees of macroporosity, as has recently been reported and discussed by our group.^23^

To quantify the degree of internal support fragmentation of a given particle, we introduced a fragmentation parameter *F* (eq 1)
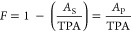
1

This corresponds to 1 minus the ratio between the area of high intensity, silica-dominant domains *A*_S_, as determined via a manually assigned threshold (Supporting Information, Section S4 and Figure S13), and the total particle area (TPA), thus yielding the sum of the areas of the polymer-dominant domains and the macropore space (*A*_P_) divided by TPA. As can be derived from the histograms of the particles’ fragmentation parameters (*F*) at different reaction stages as well as the corresponding SDs, interparticle heterogeneity is clearly evident and becomes more pronounced with the reaction progress (Figures 3D–F and Table 2). The average *A*_P_/TPA ratio (*F*_avg_) was found to increase from 0.30 at 0.8 g_PE_/g_cat_ (0.5 min) to 0.73 at 4.8 g_PE_/g_cat_ (5 min) (Table 2 and Figure 3G). In fact, this average fragmentation parameter *F*_avg_ is linearly correlated with the polymer yield in this low polymer yield regime (Figure 3H; linear relationship may not apply at higher polymer yields/larger average particle sizes).

The average fragmentation parameter of the 0.5 min prepolymerized batch (*F* = 0.30) was used as a threshold to categorize all particles of a given sample according to their respective fragmentation degrees. This allowed us to quantify the number of low-activity or “dormant” particles with *F* < 0.30 during the early reaction stages. The share of dormant catalyst particles was found to be significant after 0.5 min (52.1%) and 1 min (37.3%) of polymerization (Figure 3I).

These differences in reactivity at reaction onset partly explain the large spread in particle sizes observed at more advanced reaction stages (Figure 2). Even after 5 min of polymerization (4.8 g_PE_/g_cat_), 7.4% of the characterized particles possess *F* values smaller than 0.30, implying that they have only fragmented to a limited extent.

While the 2D analysis generally does not deliver accurate compositional data for a single particle, it is useful for extracting trends in composition and reactivity over several catalyst batches (or reaction stages) by calculating the average values for each batch. The linear relationship between *F* and the polymer yield can furthermore be used to determine the unknown polymer yield of a given catalyst batch using minimal sample amounts. In contrast to techniques such as video microscopy, which has predominantly been applied during gas-phase polymerization experiments, 2D CFM is suitable for assessing internal support fragmentation and catalyst particle activity in both gas-phase and slurry-phase prepolymerized samples, making it a useful tool for catalyst characterization and quality control.

### Assessing Interparticle Heterogeneity and Size-dependent Morphological Correlations with 3D CFM

**Figure 4:**
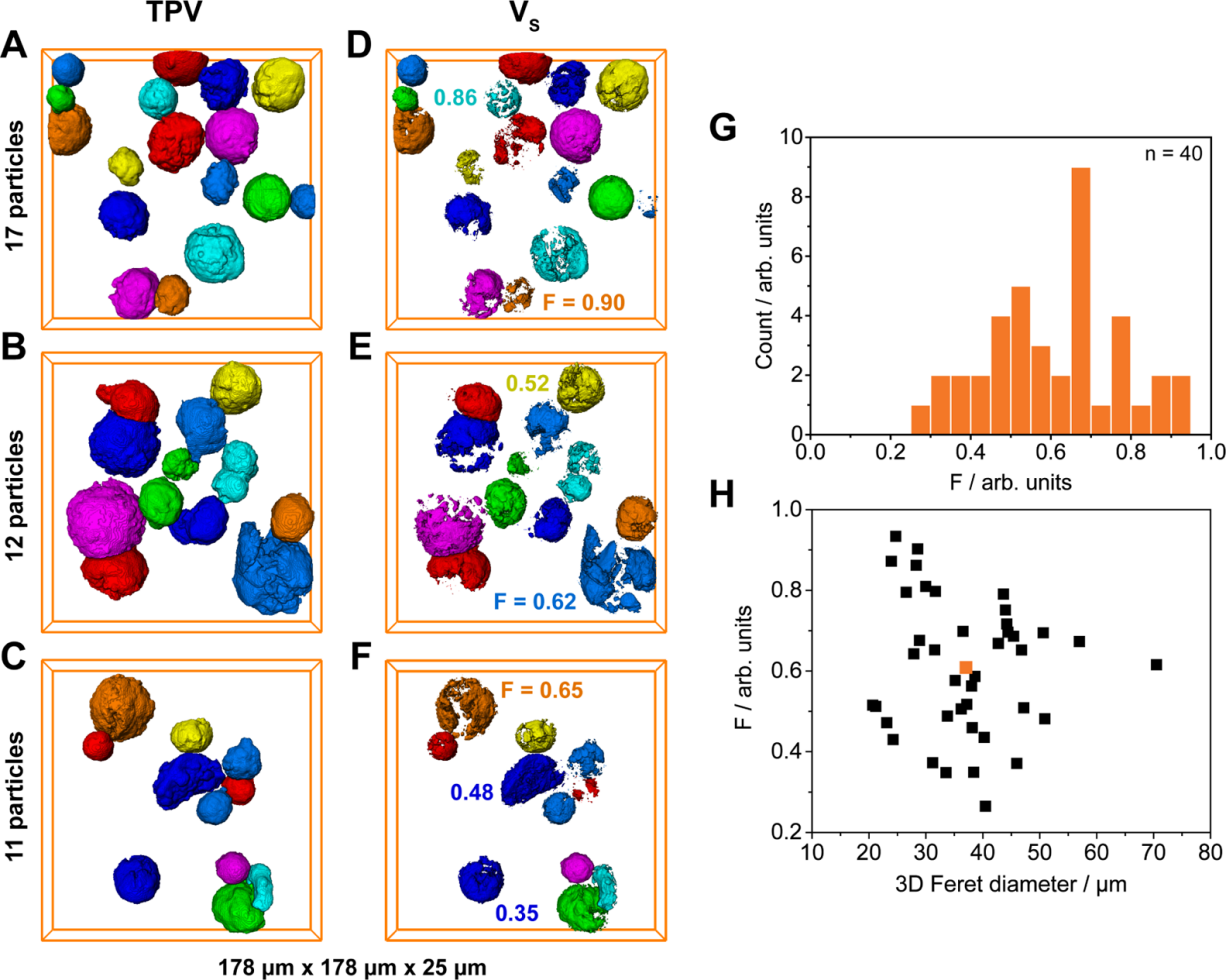
Three-dimensional CFM data acquired of 40 particles of the 1 min prepolymerized Zr/MAO/SiO_2_ catalyst (10 bar ethylene, room temperature, 2.1 g_PE_/g_cat_). (A–C) TPV of the characterized particles; (D–F) segmented high-intensity regions of the particles that represent the silica-dominant phase (*V*_S_); (G) histogram of the particles’ respective fragmentation parameters (*F* = *V*_P_/TPV); and (F) largest 3D Feret diameters of the particles plotted vs their respective fragmentation parameters *F* (average value plotted in orange).

With the 2D analysis clearly indicating the differences in morphology between individual particles, further efforts were made to extract more accurate quantitative data with 3D CFM. 40 catalyst particles of the 1 min prepolymerized batch were thus scanned over a range of 25 μm in depth (*Z*) using a step size of 0.125 μm (Movie S1). The *Z*-stacks of 2D images were consequently segmented to determine the particles’ respective volumes (i.e., total particle volume = TPV; Figure 4A–C) and treated with a manual thresholding algorithm to isolate the high-intensity regions representing the silica-dominant phase V_S_ (Figure 4D–F; Supporting Information, Section S4 and Figure S13). After a visual inspection of the reconstructed particles, the data sets were adapted to only include particles with sufficiently large volumes within the FOV. As previously observed in the 2D analysis, the particles’ internal fragmentation parameters (*F* = *V*_P_/TPV) varied significantly (*F* = 0.26–0.93, *F*_avg_ = 0.61, Figure 4G). The fragmentation parameter values of selected particles can be extracted from Figures 4D–F. By applying a k-means clustering algorithm to the data set (3 clusters with the following centroids: F = 0.37/0.52/0.75), the particles were roughly classified in relation to each other based on their F values . Out of 40 catalyst particles, 7, 13, and 20 particles displayed weak, moderate, and strong degrees of fragmentation, respectively. 29 of the 40 catalyst particles (72.5%) were found to be composed of more than 50% PE (*F* > 0.50), while only 9 catalyst particles (22.5%) contained more than 75% PE (*F* > 0.75).

In the past, inverse correlations between the particle size and catalyst activity have been reported and were generally attributed to a higher diffusion resistance in larger particles.^13,57−62^ To determine whether the morphological heterogeneity observed here is actually linked to the size of the particles, the particles’ respective fragmentation parameters (*F*) were set in relation to their largest 3D Feret diameters (Figure 4H). The 3D Feret diameter describes the distance between a pair of parallel tangential planes that confine a given object in three dimensions and is therefore a representative measure for the true particle size. The average Feret diameter of the 40 prepolymerized particles was determined as 37.1 μm (Table 3), which is comparable to the average particle size that was obtained via optical microscopy (39.8 μm, Table 1). The small deviation in value may be explained by the lower number of particles that was characterized with CFM. By plotting the particles’ *F* values against their respective Feret diameters (Figure 4H), marked differences in polymerization degree between similarly sized particles became apparent. No clear size dependency could, however, be established based on Pearson’s correlation coefficient of −0.11 (Table 3).

**Table 3:** Quantitative Data Extracted via 3D CFM and Image Processing for the 1 min Prepolymerized Zr/MAO/SiO_2_ Catalyst Batch (10 bar Ethylene)^a^

sample	*n*	*F*_avg_	SD/%	*d*_Feret,avg_/μm	PCC
1 min	40	0.61	16.8	37.1	–0.11

a *n* = total number of particles; *F*_avg_ = average fragmentation parameter *F* of all particles; SD = standard deviation of *F*; *d*_Feret,avg_ = average 3D Feret diameter; and PCC = Pearson’s correlation coefficient for *F* and *d*_Feret_.

The large spread in fragmentation parameter values (Figure 4G) leads us to believe that the structural heterogeneity of the particles’ supports (Figure S3), rather than the particle size, is the more dominant factor in controlling the monomer diffusion and, thus, the kinetics and morphological evolution of the catalyst particles during these early reaction stages. At more advanced reaction stages and under higher mass-transfer limitations, size-dependent effects may become more pronounced, as previously reported in the literature.^13,57−62^ The cross-sectional data acquired at 10 μm depth, despite yielding a far less reliable size determination, further corroborate our hypothesis that the interparticle heterogeneity is not solely attributable to the particle size (see Pearson’s correlation coefficients in Table S2 and Figure S14).

### Qualitative Interpretation of the Morphology Data

The significant inter- and intraparticle heterogeneities observed in the Zr/MAO/SiO_2_ catalyst at multiple stages of slurry-phase ethylene polymerization (Figures S3–S11 and Movie S1) confirm our conclusions from previous studies on gas-phase prepolymerized metallocenes.^19,22^ As is postulated in these recent works, the morphological evolution of an individual catalyst particle is strongly correlated to its initial support architecture and pore space configuration. These effectively determine the degree of mass transport and, thus, reaction kinetics at the single particle level. Particles with smaller, accessible support domains (higher support macroporosity and pore space interconnectivity) are expected to display a more advanced fragmentation degree than catalyst particles that are predominantly constituted by large granulates with limited macroporosity (i.e., higher mass-transfer resistance).

Interestingly, olefin polymerization is often observed to a greater extent in a specific subvolume of particles (Figures 3A–C and 4A–F). Higher accessibilities may exist for specific support domains, which could be related to their spatial arrangement within the particle. While interactions with other particles (i.e., agglomeration) or with the walls of the reactor should also be considered, their effects are expected to be limited as the reaction mixture was continuously stirred, even before ethylene addition. Low to moderate variations in fluorescence intensity among the pristine catalyst particles (Figure S3) indicate different metallocene loadings, which can potentially lead to differences in reactivity among individual particles. Tran et al. recently reported inhomogeneities in the radial distributions of a zirconocene complex and MAO in the cross sections of individual silica-supported catalyst particles.^63^ Higher concentrations of the metallocene complex in the periphery of the catalyst particles were found to lead to a more pronounced formation of fines, thus indicating correlations between the metallocene distribution, local polymerization activity, and morphology. Knoke et al. and Velthoen et al. also proposed an inhomogeneous distribution of MAO among catalyst particles as a possible cause for the variations in reactivity and morphology.^16,64^ With the Zr/MAO/SiO_2_ catalyst featuring ∼15 wt % aluminum, inter- and intraparticle heterogeneities, in terms of the distribution of MAO, should be minimal.^64^ This was confirmed with a SEM–EDX analysis (Figure S15).

The 2D and 3D data (Movie S1) representatively show that the layer-by-layer fragmentation mechanism dictates the fragmentation of the catalyst particles under the given reaction conditions (slurry-phase ethylene polymerization, 10 bar). A synergy with, in this case, the more subdued sectioning mechanism is also apparent. The contribution of this mechanism is, however, limited to particles with significant mass-transfer limitations and is responsible for the formation of more extensive cracks in the catalyst support.

## Conclusions

Due to its fast measurement times and comparatively large FOV, laboratory-based CFM represents an efficient, noninvasive diagnostic tool to obtain quantitative information on the relative composition and morphology of pristine as well as prepolymerized olefin polymerization catalyst particles, both in two and three dimensions. By delivering statistically more relevant mechanistic insights into the morphological evolution of a given catalyst system, it represents an attractive complementary method to hard X-ray-based nanotomography techniques, which are often limited by their FOV, long measurement periods, and elaborate data reconstruction and analysis.

Our studies on a silica-supported zirconocene-based catalyst material revealed large differences in reactivity between individual catalyst particles at the onset of ethylene polymerization. The dormant behavior of the selected catalyst particles during the early reaction stages leads to delays in particle growth, which partly accounts for size-based differences in the final polymer product. The acquired 2D and 3D data, collected on a representative number of catalyst particles, furthermore revealed significant inter- and intraparticle heterogeneities during the early stages of polymerization. A strong correlation of fragmentation with the support and pore space architecture of the individual catalyst particles is apparent from our investigations on samples that were prepolymerized to low polymer yields.

In general, the linear correlation between the polymer yield and the fragmentation parameter *F* can be exploited to determine the unknown polymer yields of samples. Provided that several reaction stages are evaluated in three dimensions, the polymer yield of individual catalyst particles could be derived from their respective fragmentation parameters. This represents a novel approach to estimate the activities of individual polymerization catalyst particles.

By means of rational catalyst design and material-specific staining procedures,^41,43^ the methodology can also be extended to other supported olefin polymerization catalysts, where morphological screening is desired at high sample throughput. In fact, the approach is applicable to any type of macroporous catalyst system, where representative structural and chemical insights are desired at the single catalyst particle level. By using autofluorescent metallocene-based catalysts (several metallocenes display fluorescence), or other support-stained polymerization catalysts, in combination with fluorescence-based particle screening,^65^ it may also be possible to sort pristine polymerization catalyst particles according to their support structure and metallocene concentration. Hence, by minimizing this disparity between catalyst particles, a greater control over a given polymerization catalyst’s activity and morphological evolution may be achieved. Finally, with machine learning gaining momentum, fully automated image segmentation and postprocessing could greatly improve the efficiency of the 3D data analysis.

## Supporting Information Available

Information on the preparation, testing, prepolymerization, and characterization of the silica-supported metallocene-based catalyst samples; extensive collection of CFM and FIB–SEM data; and detailed description of the image processing, segmentation, and data analysis that were applied to the collected CFM data (PDF)
Scan of 40 catalyst particles of the 1 min prepolymerized batch over a range of 25 μm in depth using a step size of 0.125 μm (MPG)

## Supplementary Material

ja2c09159_si_001.pdf

ja2c09159_si_002.mpg
