# Supplementary material for: Advancing the Compositional Analysis of Olefin Polymerization Catalysts with High-Throughput Fluorescence Microscopy
Source: J Am Chem Soc. 2022 Nov 8;144(46):21287–94. doi: 10.1021/jacs.2c09159 (PMC9706558; doi:10.1021/jacs.2c09159)
Supplement: Supplementary file 1 — ja2c09159_si_001.pdf [file ja2c09159_si_001.pdf]

Supporting Information

Advancing the Compositional Analysis of Olefin Polymerization Catalysts with High-Throughput Fluorescence Microscopy

Maximilian J. Werny,[a,b] Kirsten B. Siebers,[a] Nicolaas H. Friederichs,[c] Coen Hendriksen,[c] Florian Meirer,[a,*] and Bert M. Weckhuysen[a,*]

[a] Inorganic Chemistry and Catalysis group, Institute for Sustainable and Circular Chemistry and Debye Institute for Nanomaterials Science, Utrecht University, Universiteitsweg 99, 3584 CG Utrecht (The Netherlands)

[b] Dutch Polymer Institute (DPI), P.O. Box 902, 5600 AX Eindhoven (The Netherlands)

[c] SABIC Technology Center, Urmonderbaan 22, 6167 RD Geleen (The Netherlands)

Table of Contents

Experimental Procedures

Section S1: Catalyst synthesis

Section S2: Catalyst pre-polymerization

Table S1. Polyethylene (PE) yields in g_{PE}/g_{cat} catalyst from the slurry-phase pre-polymerization of the Zr/MAO/SiO_2 catalyst at 10 bar ethylene pressure and room temperature.

Section S3: Catalyst characterization

A. Optical microscopy

B. Confocal fluorescence microscopy

C. Focused ion beam - scanning electron microscopy

D. Scanning electron microscopy-energy dispersive X-ray spectroscopy

Table S2. Settings used for the 2D and 3D confocal fluorescence microscopy (CFM) measurements of the Zr/MAO/SiO_2 catalyst.

Section S4: Image processing and segmentation

Table S3. Polyethylene (PE) to silica (SiO_2) volume ratios, as derived from the yields of the 0.5 min, 1 min and 5 min pre-polymerized samples [assuming δ(PE) = 0.95 g/cm^3, δ(SiO_2) = 1.60–1.90 g/cm^3, denoted as V_{PE}/V_{SiO2} (yield)], compared to the volume ratios derived from the 2D and 3D confocal fluorescence microscopy (CFM) data analysis.

Table S4. Percentage of partial particle cross-sections per characterized reaction stage as measured by 2D confocal fluorescence microscopy (CFM).

Supporting Figures and Tables

Figure S1. Emission spectrum of the pristine Zr/MAO/SiO_2 catalyst at 488 nm excitation (no dichroics).

Figure S2. 2D confocal fluorescence microscopy (CFM) images recorded of SiO2 and MAO/SiO2 particles (1024 x 1024 pixels, dichroics: 405 nm/488 nm/561 nm/640 nm). The brightness of the images has been increased significantly to visualize the non-florescent particles.

Figure S3. A selection of 2D confocal fluorescence microscopy (CFM) images recorded of the pristine Zr/MAO/SiO_2 catalyst (1024 x 1024 pixels, dichroics: 405 nm/488 nm/561 nm/640 nm).

Figure S4. A selection of 2D confocal fluorescence microscopy (CFM) images recorded of the 0.5 min pre-polymerized Zr/MAO/SiO_2 catalyst (1024 x 1024 pixels, dichroics: 405 nm/488 nm/561 nm/640 nm).

Figure S5. A selection of 2D confocal fluorescence microscopy (CFM) images recorded of the 1 min pre-polymerized Zr/MAO/SiO_2 catalyst (1024 x 1024 pixels, dichroics: 405 nm/488 nm/561 nm/640 nm).

Figure S6. A selection of 2D confocal fluorescence microscopy (CFM) images recorded of the 5 min pre-polymerized Zr/MAO/SiO_2 catalyst (1024 x 1024 pixels, dichroics: 405 nm/488 nm/561 nm/640 nm).

Figure S7. A selection of 2D confocal fluorescence microscopy (CFM) images recorded of the 15 min pre-polymerized Zr/MAO/SiO_2 catalyst (1024 x 1024 pixels, dichroics: 405 nm/488 nm/561 nm/640 nm).

Figure S8. Scanning electron microscopy (SEM) images displaying the horizontal cross-sections of Zr/MAO/SiO_2 catalyst particles that were pre-polymerized in slurry-phase for 0.5 min at 10 bar ethylene (room temperature).

Figure S9. Scanning electron microscopy (SEM) images displaying the horizontal cross-sections of Zr/MAO/SiO_2 catalyst particles that were pre-polymerized in slurry-phase for 1 min at 10 bar ethylene (room temperature). Additional images can be found in reference [3].

Figure S10. Scanning electron microscopy (SEM) images displaying the horizontal cross-sections of Zr/MAO/SiO_2 catalyst particles that were pre-polymerized in slurry-phase for 5 min at 10 bar ethylene (room temperature).

Figure S11. Scanning electron microscopy (SEM) images displaying the horizontal cross-sections of Zr/MAO/SiO_2 catalyst particles that were pre-polymerized in slurry-phase for 15 min at 10 bar ethylene (room temperature).

Figure S12. Line scan analysis performed on a single particle of the pristine Zr/MAO/SiO_2 catalyst using the 10%–90% criterion. A resolution of 374.9 nm was determined from the edge profile.

Figure S13. Overview of the image processing applied to the 2D and 3D confocal fluorescence microscopy (CFM) data sets. The 16-bit greyscale images (1) were filtered with a non-local means filter and masked with the TPAs/TPVs (2) of the particles. After collectively normalizing the masked 2D and 3D CFM data (3), the data sets were segmented into high-and low-intensity regions using manually defined thresholds. The high-intensity domains (4) represent a silica-dominant phase (V_S).

Figure S14. Largest Feret diameters of the 0.5 min (blue), 1 min (red) and 5 min (green) pre-polymerized Zr/MAO/SiO_2 catalyst particles that were characterized with 2D confocal fluorescence microscopy (CFM), plotted versus their respective fragmentation parameters (F).

Figure S15. Scanning electron microscopy-energy dispersive X-ray spectroscopy (SEM-EDX) data recorded of three catalyst particles of the pristine Zr/MAO/SiO_2 catalyst. A: SEM-EDX data of a full catalyst particle acquired with a FEI Helios NanoLab G3 UC scanning electron microscope at 10 keV, B: SEM-EDX data of two particle cross-sections acquired with a ZEISS GeminiSEM 450 at 5 keV. The EDX images display the distribution of zirconium (Zr) and chlorine (Cl), representative for the zirconocene complex, as well as aluminum (Al), which is representative for the co-catalyst methylaluminoxane (MAO).

References

Supporting Videos

Video S1. Z-stacks of processed 2D confocal fluorescence microscopy images that were acquired of the pre-polymerized silica-supported zirconocene-based catalyst (1 min, 10 bar, 2.1 g_{PE}/g_{cat}). All images were filtered with a non-local means filter, masked using the total particle areas (TPAs) and visualized using a grayscale color map. The figures 4A and 4C, 4B and 4D, and 4C and 4F in the main text are based on Scans A, B and C, respectively. The histogram of the 2D slices was adjusted to obtain better contrast, hence leading to the absence of individual low intensity pixels.

Experimental Procedures

Section S1: Catalyst synthesis

The investigated olefin polymerization catalysts were supplied by Saudi Basic Industries Corporation (SABIC). All synthetic procedures were performed under inert N_2 atmosphere.

The zirconocene-based catalyst material Zr/MAO/SiO_2 was prepared according to a procedure mentioned in previous publications.[1–3] 5 g of ES757 silica (PQ Corporation, D_{50} = 25.0 µm, S_{BET} = 295 m²/g, V_{Pore} = 1.6 mL/g), calcined for 4 h at 600 °C, were impregnated with a solution of the corresponding bis-indenyl metallocene complex (Zr = 2,2'-biphenylene-bis-2-indenyl $ZrCl_2$, 0.244 mmol) and methylaluminoxane (MAO, 30 wt%, Chemtura; 7.6 mL; Al/M molar ratio = 150) in dried toluene (15.6 mL, Braun solvent purification system) at room temperature. The resulting slurry was dried with a stream of N_2 at room temperature for 20 h to produce a free-flowing powder. The zirconocene-based catalyst sample (S_{BET} = 244 m²/g, V_{Pore} = 0.67 mL/g) possesses a weight loading of ~0.30 wt% Zr as determined via X-ray fluorescence (XRF) analysis.

Section S2: Catalyst pre-polymerization

The silica-supported zirconocene-based catalyst Zr/MAO/SiO_2 was pre-polymerized at room temperature in slurry-phase at 10 bar ethylene pressure in a Parr autoclave set-up under stirring (570 rpm). In a first step, the autoclave was loaded inside a nitrogen glovebox. Approximately 10 mL heptane and 3 µL triisobutylaluminum (TiBA, scavenger) were added to 10 mg of catalyst powder in a glass reactor. This glass reactor was then placed inside the autoclave. After removal from the glovebox, the autoclave was pressurized for ~ 10 s under continuous stirring to reach the desired pressure. The inlet valve was then closed. The period of pressurization is included in the total polymerization time (Table S1). To terminate the reaction, the valves of the autoclave were opened, and the formed polymer immediately removed from the glass reactor. The polymer was dried under air flow and weighed. Polymer yields were calculated by subtracting the initial mass of catalyst from the final mass of the polyethylene-catalyst composite.

Table S1. Polyethylene (PE) yields in g_{PE}/g_{cat} from the slurry-phase pre-polymerization of the Zr/MAO/SiO_2 catalyst at 10 bar ethylene pressure and room temperature.

Time / min	0.5	1	5	15
Yield / g_{PE}/g_{cat}	0.8	2.1	4.8	18.5

Section S3: Catalyst characterization

A. Optical microscopy was employed to determine the particle size distribution, average particle sizes and D_{50} values of the pristine and pre-polymerized catalyst batches. A Zyla camera, installed on a Nikon A1 confocal fluorescence microscope, was used. The analysis of the acquired images was performed with ImageJ. For each sample batch, the largest 2D Feret diameters of 200 particles were assessed. The 2D Feret diameter describes the distance between a pair of parallel tangential lines that confine a given particle in 2D.

B. Confocal fluorescence microscopy (CFM) was performed using a Nikon A1 confocal microscope, configured with an Eclipse Ti2-E inverted microscope body, at 488 nm excitation. The system is equipped with a pin hole to filter out-of-focus light and suitable dichroic mirrors (405 nm/488 nm). A Nikon oil immersion objective (Nikon CFI Plan Apo Lambda 60x Oil, NA = 1.4) was employed in combination with a Nikon type F immersion oil for index matching (refractive index oil = 1.518, refractive index HDPE ≈ 1.51–1.54[4–6], refractive index amorphous silica ≈1.45–1.47[7–9]). In terms of sample preparation, the pre-polymerized, air-exposed samples were placed on top of a microscopy slide, immersed in oil and covered with a slide of 170 µm thickness. All 2D CFM images were acquired at a focal depth of 10 µm. To obtain the data for the 3D reconstructions, Z-stacks of multiple 2D CFM images were acquired at a step size of 0.125 µm. By applying a scan size of 2048 x 2048 pixels to a 178.0 µm x 178.0 µm field of view (pixel size = 86.9 nm, Nyquist sampling, theoretical optical resolution ≈ 0.20 µm), a lateral resolution of ~470 nm was obtained. The resolution was determined based on 15 line profiles that were fitted over well-defined features in the filtered 2D CFM images of the pristine Zr/MAO/SiO_2 catalyst following a 10%–90% criterion[10,11] (line scan analysis, Figure S12). More detailed information on the measurement settings can be found in Table S2. All overview images in the Supporting Information (Figures S2–S7) were measured using 1024 x 1024 pixels and a field of view of 294.6 µm x 294.6 µm and were only used to assess the fragmentation of different samples qualitatively.

Table S2. Settings used for the 2D and 3D confocal fluorescence microscopy (CFM) measurements of the Zr/MAO/SiO_2 catalyst.

Setting	Value
Excitation wavelength / nm	488
Laser power / %	7.5
Spectral detection range / nm	508–748
Si grating resolution / nm	10
Scan size / pixels	2048 x 2048
Frames per second	1/32
Averaging	No averaging
Pinhole size / µm	35.8
Si HV	180

C. Focused ion beam-scanning electron microscopy (FIB-SEM) was performed on individual catalyst particles using a FEI Helios NanoLab G3 UC scanning electron microscope following procedures from literature.[1,12] The samples were first mounted onto double-sided adhesive, conductive carbon tape, which was attached to an aluminum SEM stub. A Cressington 208HR sputter coater was used to apply a Pt coating of ~ 6 nm thickness. Using a 45° angled SEM stub at different stage tilt angles, the particles were cut horizontally to the surface. The cross-sectional images were acquired in backscattered electron (BSE) mode at 2 kV and 0.1 nA using a Through the Lens Detector (TLD) and an immersion lens.

D. **Scanning electron microscopy-energy dispersive X-ray spectroscopy (SEM-EDX)** was performed with a FEI Helios NanoLab G3 UC scanning electron microscope at 10 keV and a ZEISS GeminiSEM 450 at 5 keV. The latter was used to characterize particle cross-sections that were accessed following the above-stated FIB procedure.

Section S4: Image processing and segmentation

Post processing of the 2D confocal fluorescence microscopy (CFM) images and visualization of the reconstructed catalyst particles was performed using self-developed code written in MATLAB™ and the Avizo™ software package by Thermo Fisher Scientific Inc.

In a first step, the 2D CFM images (.nd2 files) were exported as 16-bit greyscale TIF images using MATLAB™. All images were subsequently filtered with a non-local means filter. Depending on the nature of the data (2D/3D), the total particle areas (TPAs) or total particle volumes (TPVs) of the particles were determined. The TPV is defined as the total volume of a particle including matter and pores. Particles that were in contact with each other were separated manually in Avizo™. The particle areas or volumes and their corresponding largest 2D or 3D Feret diameters were then calculated. After masking the original images with their corresponding TPAs or TPVs, the 2D and 3D data sets were collectively normalized (value range of 0–255). An automated thresholding procedure (threshold value: 2D = 36, 3D = 24) was applied to segment the high intensity regions in the collected 2D and 3D CFM data sets. All thresholds were chosen based on visual inspection. Due to the high degree of intermixing of support and polymer phase as well as resolution limitations, the high intensity regions correspond to the sum of pure silica and silica-dominant mixed phase and are referred to as silica-dominant phase (A_S/V_S). The low intensity regions, on the other hand, represent a combination of polyethylene, polyethylene-dominant mixed phase and macropore space and are collectively denoted as V_P. A comparison of the $V_{PE+macropores}/V_{SiO2}$ volume ratios extracted from the 2D and 3D CFM data to the PE yield-derived volume ratios revealed the PE phase to be significantly underestimated (Table S3). This can theoretically be addressed by choosing a higher threshold value during segmentation. However, higher threshold values only result in a sub-optimal overlap of the segmented regions with the high intensity domains of the particles. Given that the fluorescence intensity decreases strongly, even after 0.5 min of polymerization (Figure S3), we believe that a large amount of the formed PE phase goes undetected due to resolution limitations. This affects any quantification, as is apparent below.

Table S3. Polyethylene (PE) to silica (SiO_2) volume ratios, as derived from the yields of the 0.5 min, 1 min and 5 min pre-polymerized samples [assuming δ(PE) = 0.95 g/cm^3, δ(SiO_2) = 1.60–1.90 g/cm^3, denoted as V_{PE}/V_{SiO2} (yield)], compared to the volume ratios derived from the 2D and 3D confocal fluorescence microscopy (CFM) data analysis.

**Yield / g_{PE}/g_{cat}**	**V_{PE}/V_{SiO2} (yield)**	**$V_{PE+macropores}/V_{SiO2}$ (2D CFM)**	**$V_{PE+macropores}/V_{SiO2}$ (3D CFM)**
0.8	1.35–1.60	0.39	/
2.1	3.54–4.20	0.68	1.49
4.8	8.08–9.60	2.42	/

Low to moderate differences in fluorescence intensity can generally be observed between individual particles in the pristine catalyst material (Figure S2). Due to the strong decrease in fluorescence intensity upon the formation of non-fluorescent

polymer (Figures S3–S6), these differences were found to have only limited influence on the segmentation of the data. The accuracy of the data analysis was ensured by measuring a large number of particles per reaction stage.

In the 2D data, areas constituting less than approximately 80% of the total approximated area of a given particle's cross-section were categorized as partial cross-sections. As is demonstrated in Table S4, the percentage of partial cross-sections is low for all reaction stages.

Table S4. Percentage of partial particle cross-sections per characterized reaction stage as measured by 2D confocal fluorescence microscopy (CFM).

Time / min	0.5	1	5
**Percentage of partial cross-sections / %**	7.4	6.2	5.9

Particles that were insufficiently imaged in 3D were excluded from the data analysis. This guaranteed an accurate size determination in 3D. Agglomerated particles that could not be clearly separated based on the fluorescence images were separated manually to determine their approximate size (see red and blue particles in Figure 4B, main text).

Supporting Figures and Tables

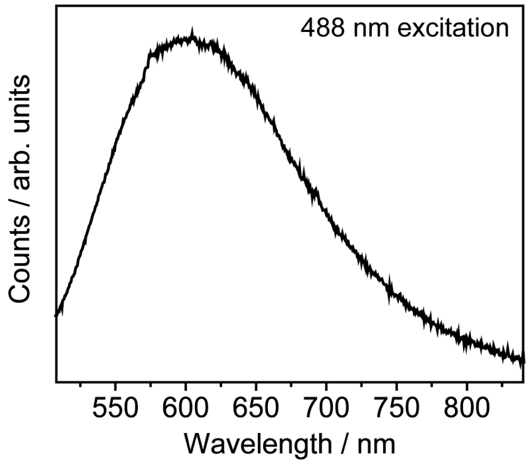

Figure S1. Emission spectrum of the pristine Zr/MAO/SiO_2 catalyst at 488 nm excitation (no dichroics).

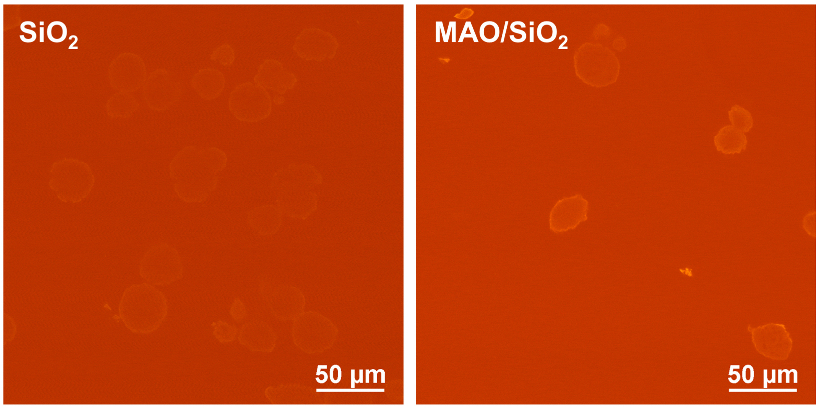

Figure S2. 2D confocal fluorescence microscopy (CFM) images recorded of SiO_2 and MAO/SiO_2 particles (1024 x 1024 pixels, dichroics: 405 nm/488 nm/561 nm/640 nm). The brightness of the images has been increased significantly to visualize the non-fluorescent particles.

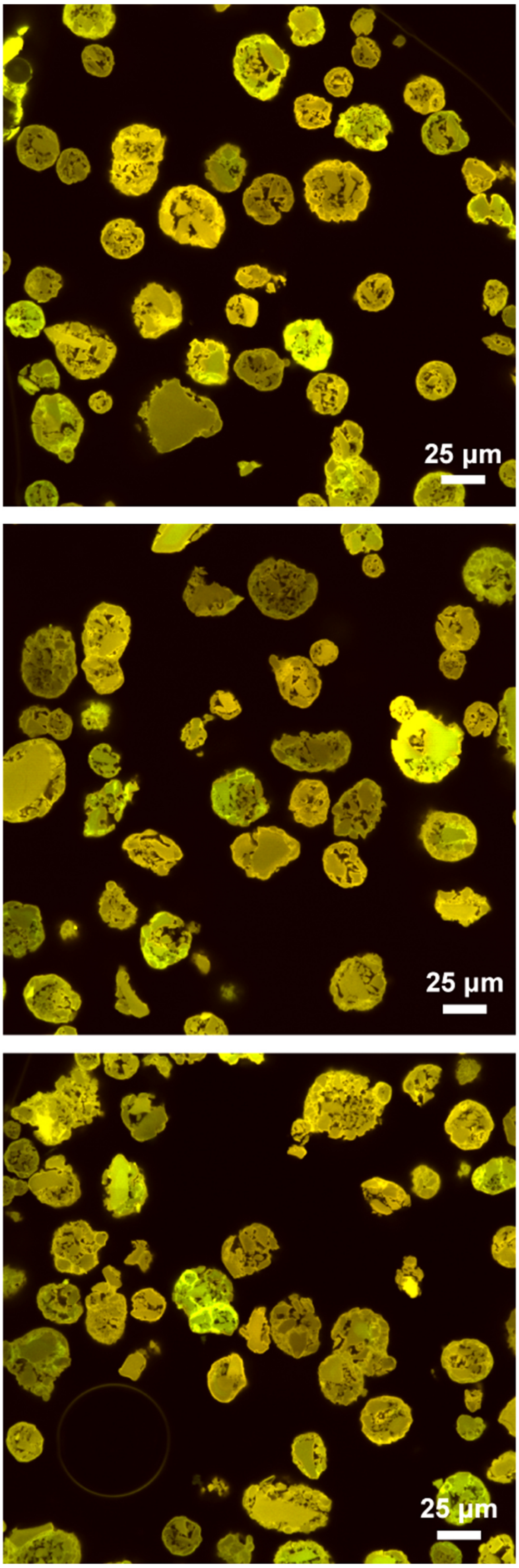

Figure S3. A selection of 2D confocal fluorescence microscopy (CFM) images recorded of the pristine Zr/MAO/SiO_2 catalyst (1024 x 1024 pixels, dichroics: 405 nm/488 nm/561 nm/640 nm).

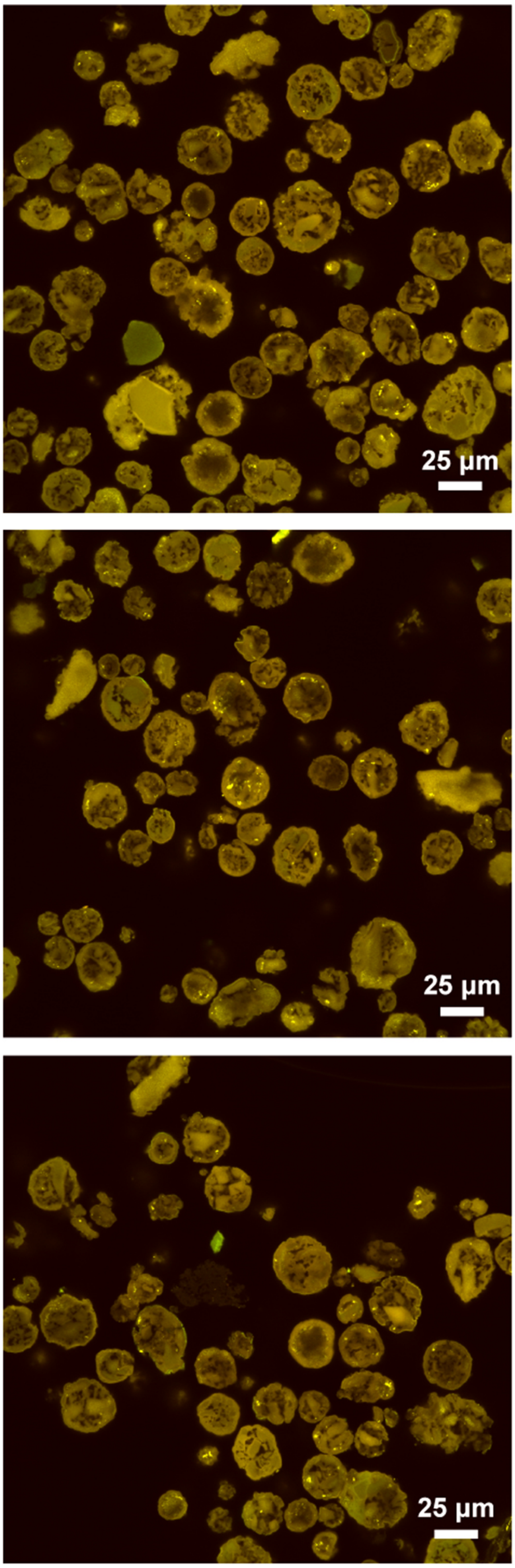

Figure S4. A selection of 2D confocal fluorescence microscopy (CFM) images recorded of the 0.5 min pre-polymerized $Zr/MAO/SiO_2$ catalyst (1024 x 1024 pixels, dichroics: 405 nm/488 nm/561 nm/640 nm).

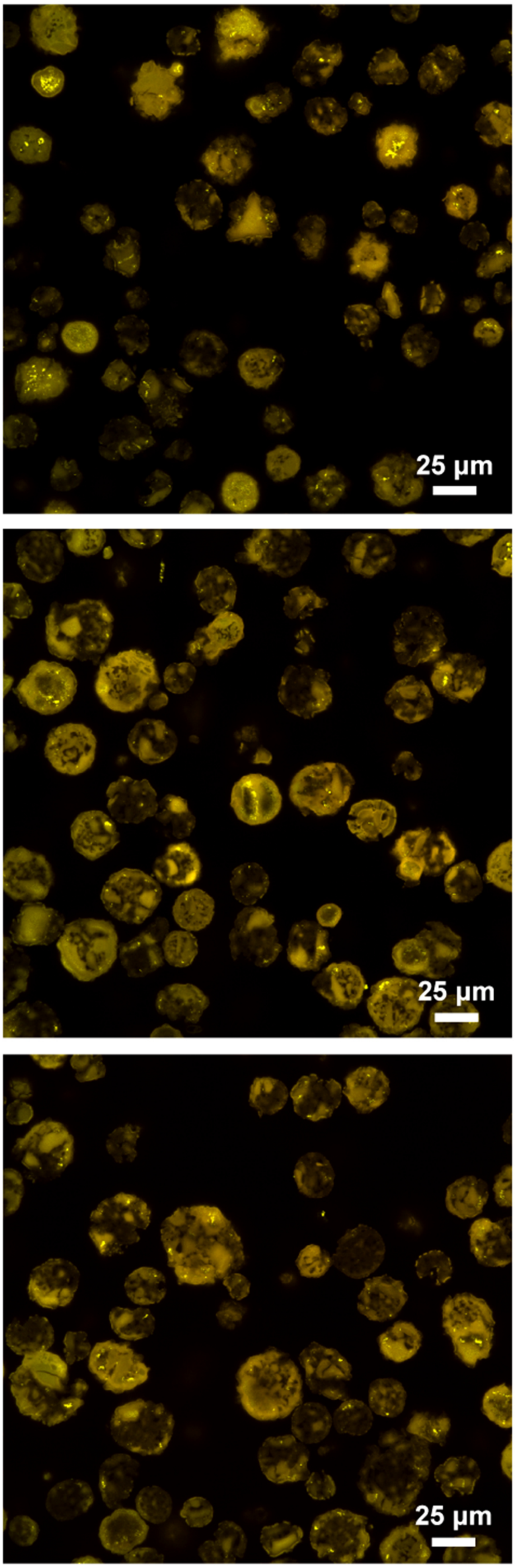

Figure S5. A selection of 2D confocal fluorescence microscopy (CFM) images recorded of the 1 min pre-polymerized Zr/MAO/SiO_2 catalyst (1024 x 1024 pixels, dichroics: 405 nm/488 nm/561 nm/640 nm).

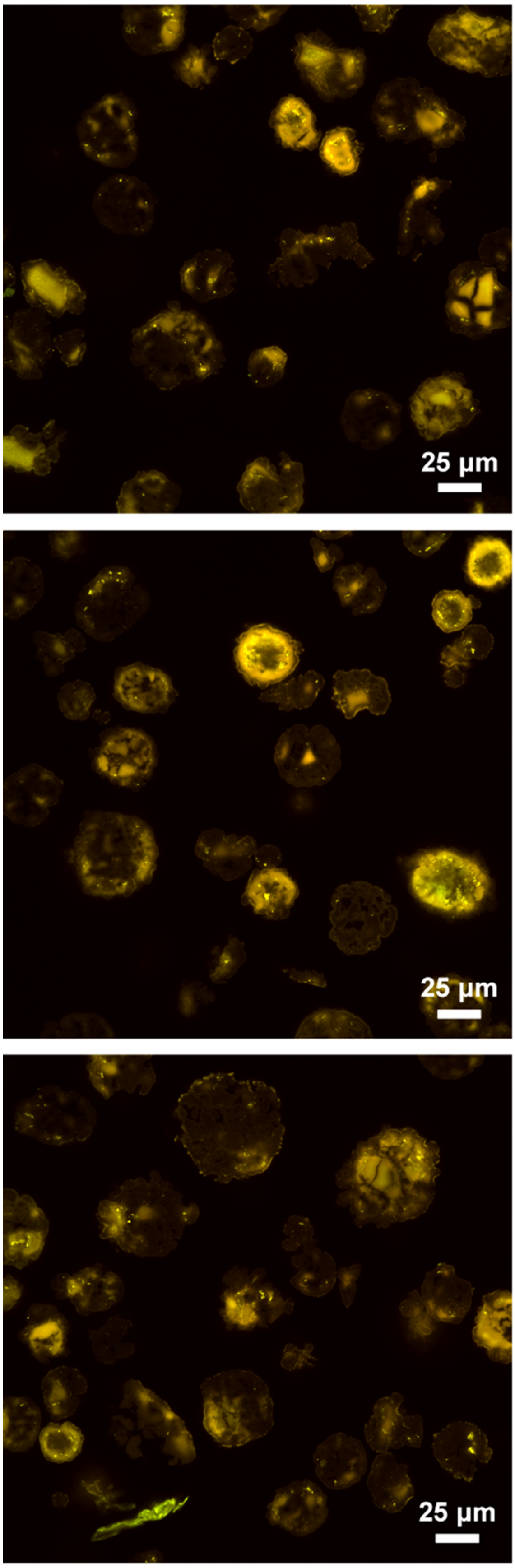

Figure S6. A selection of 2D confocal fluorescence microscopy (CFM) images recorded of the 5 min pre-polymerized Zr/MAO/SiO_2 catalyst (1024 x 1024 pixels, dichroics: 405 nm/488 nm/561 nm/640 nm).

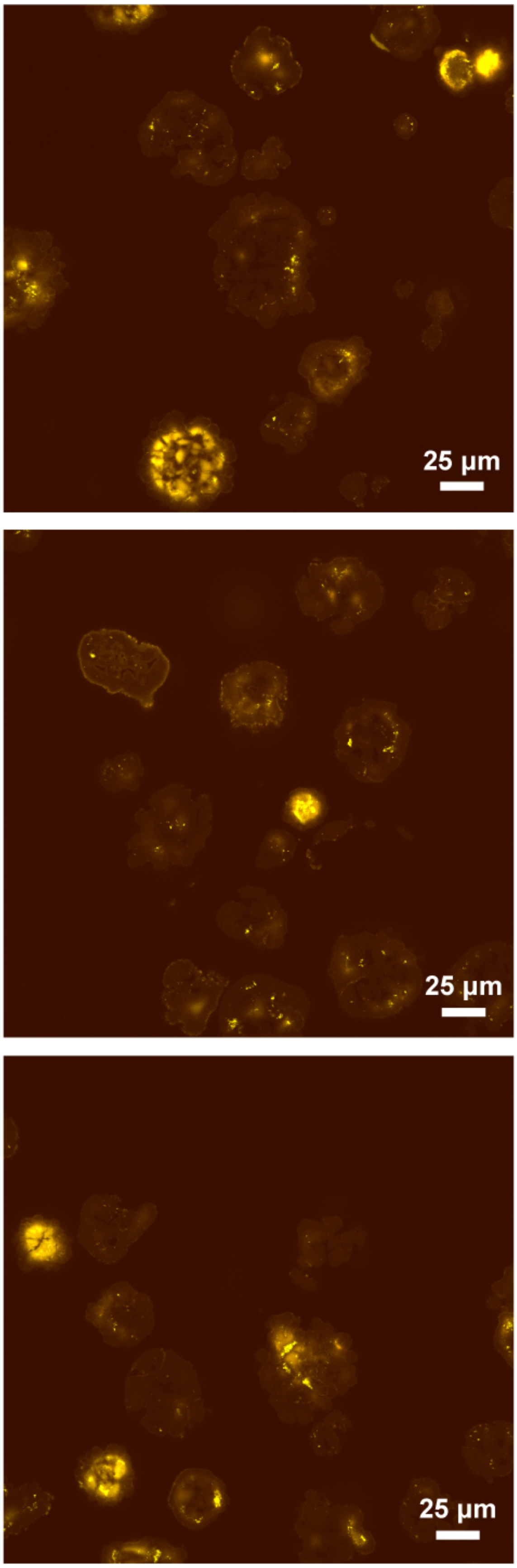

Figure S7. A selection of 2D confocal fluorescence microscopy (CFM) images recorded of the 15 min pre-polymerized Zr/MAO/SiO_2 catalyst (1024 x 1024 pixels, dichroics: 405 nm/488 nm/561 nm/640 nm). The brightness of the images was increased for better visualization.

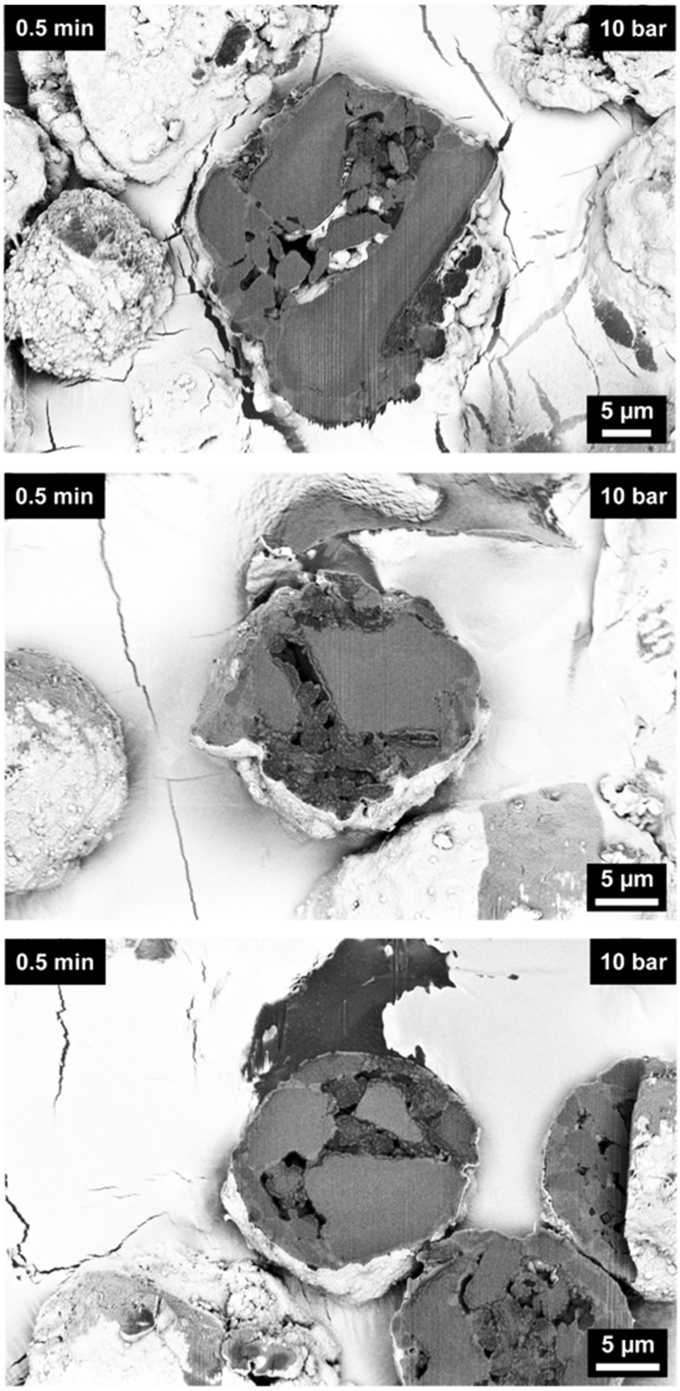

Figure S8. Scanning electron microscopy (SEM) images displaying the horizontal cross-sections of $Zr/MAO/SiO_2$ catalyst particles that were pre-polymerized in slurry-phase for 0.5 min at 10 bar ethylene (room temperature).

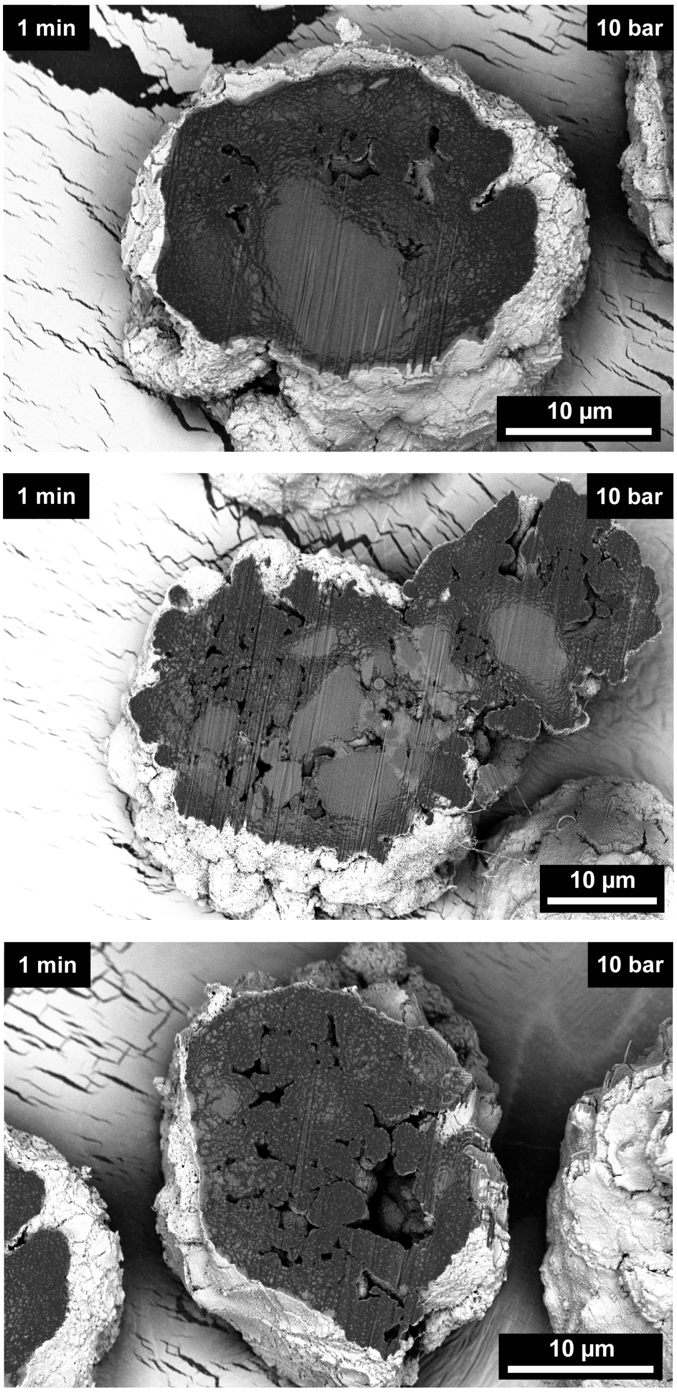

Figure S9. Scanning electron microscopy (SEM) images displaying the horizontal cross-sections of Zr/MAO/SiO_2 catalyst particles that were pre-polymerized in slurry-phase for 1 min at 10 bar ethylene (room temperature). Additional images can be found in reference [3].

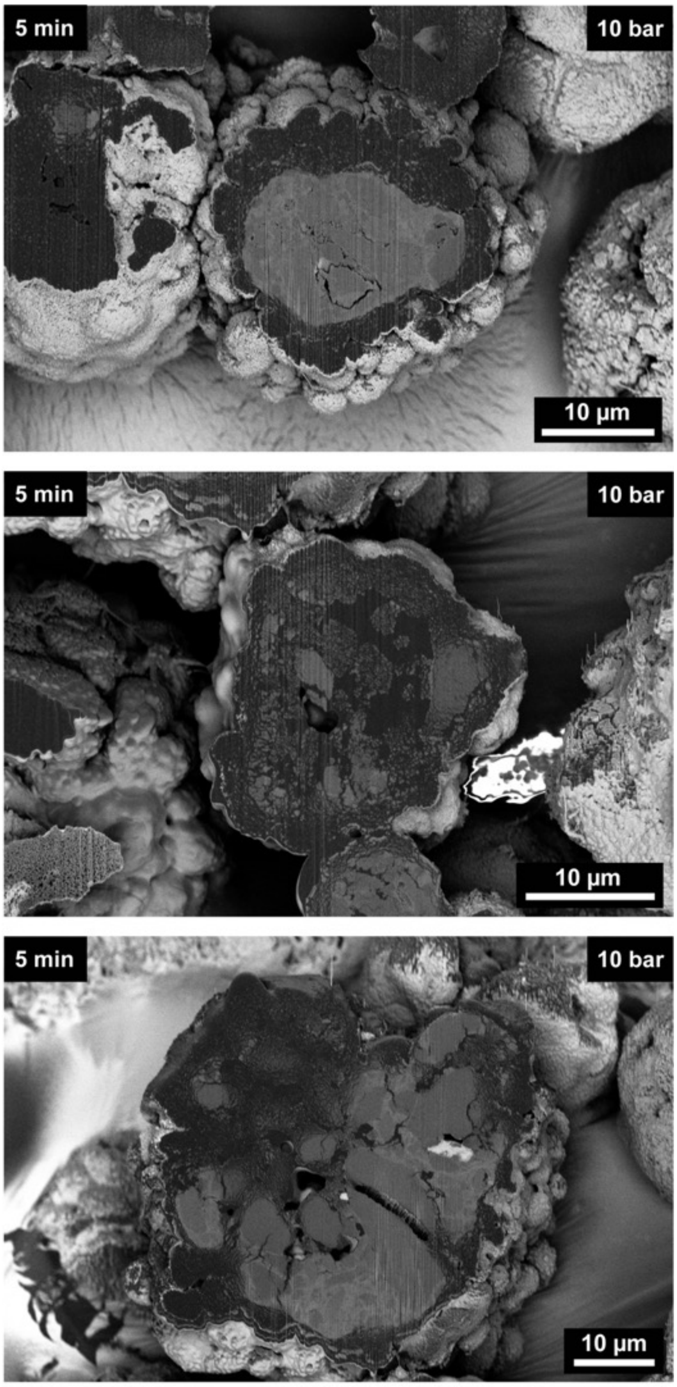

Figure S10. Scanning electron microscopy (SEM) images displaying the horizontal cross-sections of Zr/MAO/SiO_2 catalyst particles that were pre-polymerized in slurry-phase for 5 min at 10 bar ethylene (room temperature).

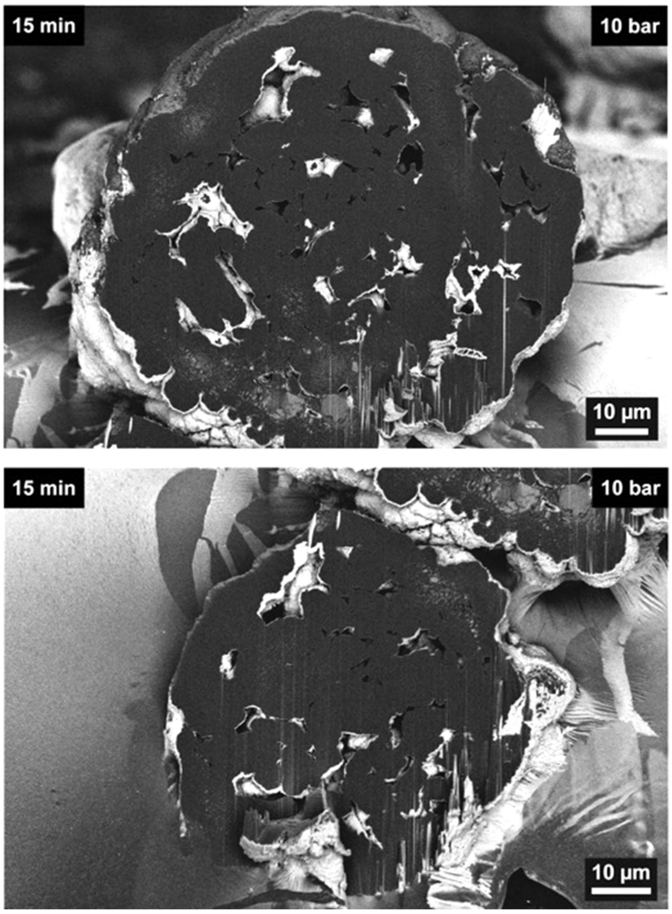

Figure S11. Scanning electron microscopy (SEM) images displaying the horizontal cross-sections of Zr/MAO/SiO_2 catalyst particles that were pre-polymerized in slurry-phase for 15 min at 10 bar ethylene (room temperature).

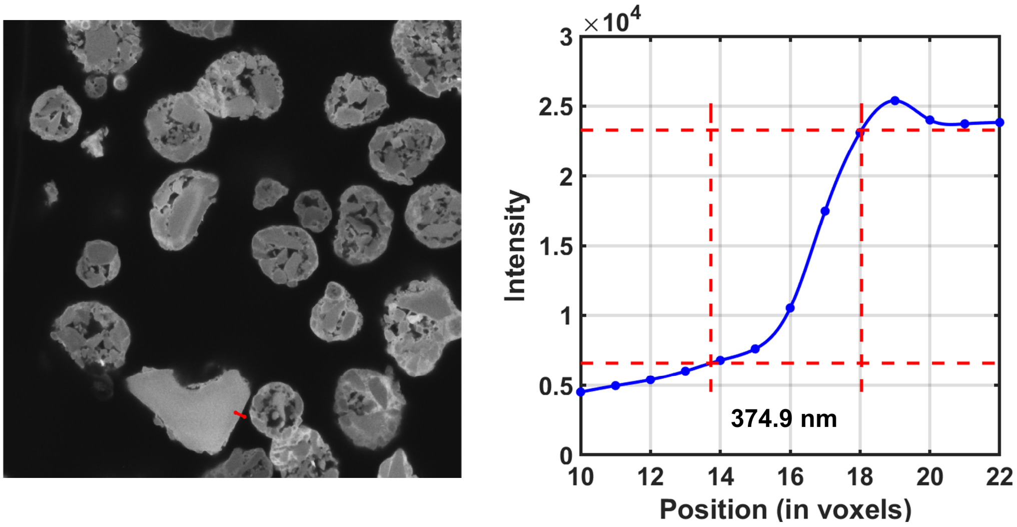

Figure S12. Line scan analysis performed on a single particle of the pristine Zr/MAO/SiO_2 catalyst using the 10%–90% criterion. A resolution of 374.9 nm was determined from the edge profile.

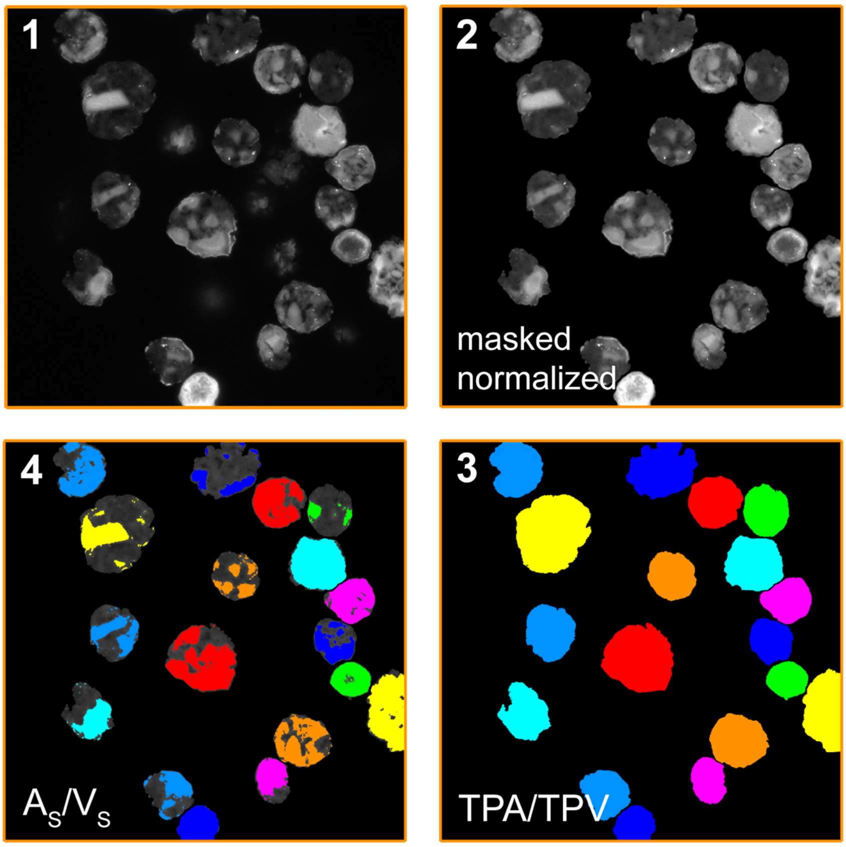

Figure S13. Overview of the image processing applied to the 2D and 3D confocal fluorescence microscopy (CFM) data sets. The 16-bit greyscale images (1) were filtered with a non-local means filter and masked with the TPAs/TPVs (2) of the particles. After collectively normalizing the masked 2D and 3D data (3), the data sets were segmented into high-and low-intensity regions using manually defined thresholds. The high-intensity regions (4) represent silica-dominant domains (A_S/V_S).

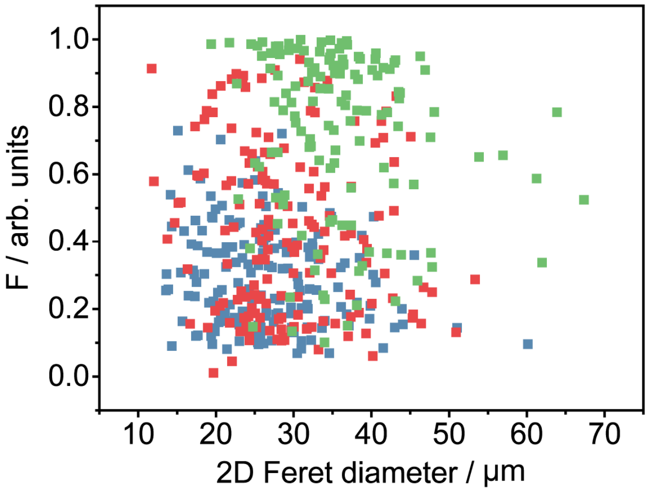

Figure S14. Largest Feret diameters of the 0.5 min (blue), 1 min (red) and 5 min (green) pre-polymerized $Zr/MAO/SiO_2$ catalyst particles that were characterized with 2D confocal fluorescence microscopy (CFM), plotted versus their respective fragmentation parameters (F).

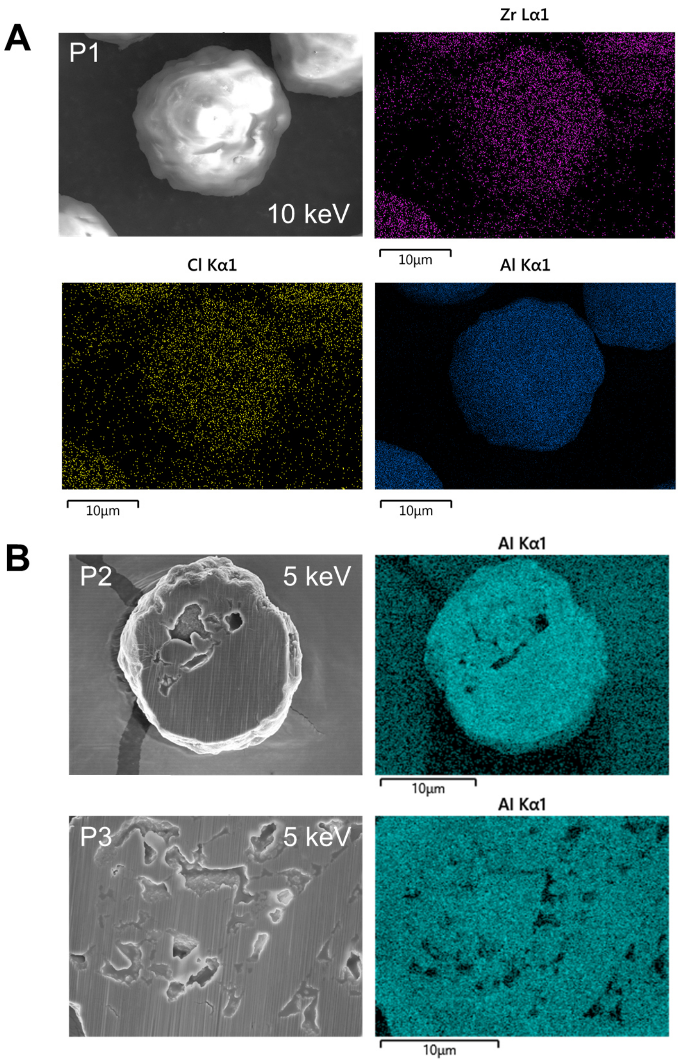

Figure S15. Scanning electron microscopy-energy dispersive X-ray (SEM-EDX) spectroscopy data recorded of three particles of the pristine Zr/MAO/SiO_2 catalyst. A: SEM-EDX data of a full catalyst particle acquired with a FEI Helios NanoLab G3 UC scanning electron microscope at 10 keV, B: SEM-EDX data of two particle cross-sections acquired with a ZEISS GeminiSEM 450 at 5 keV. The EDX images display the distribution of zirconium (Zr) and chlorine (Cl), representative for the zirconocene complex, as well as aluminum (Al), which is representative for the co-catalyst methylaluminoxane (MAO).

Supporting Videos

Video S1. Z-stacks of processed 2D confocal fluorescence microscopy images that were acquired of the pre-polymerized silica-supported zirconocene-based catalyst (1 min, 10 bar, 2.1 g_{PE}/g_{cat}). All images were filtered with a non-local means filter, masked using the total particle areas (TPAs) and visualized using a grayscale color map. The figures 4A and 4C, 4B and 4D, and 4C and 4F in the main text are based on Scans A, B and C, respectively. The histogram of the 2D slices was adjusted to obtain better contrast, hence leading to the absence of individual low intensity pixels.

References

(1) Werny, M. J.; Zarupski, J.; ten Have, I. C.; Piovano, A.; Hendriksen, C.; Friederichs, N. H.; Meirer, F.; Groppo, E.; Weckhuysen, B. M. Correlating the Morphological Evolution of Individual Catalyst Particles to the Kinetic Behavior of Metallocene-Based Ethylene Polymerization Catalysts. *JACS Au* **2021**, *1*, 1996–2008.

(2) Werny, M. J.; Valadian, R.; Lohse, L. M.; Robisch, A.-L.; Zanoni, S.; Hendriksen, C.; Weckhuysen, B. M.; Meirer, F. X-Ray Nanotomography Uncovers Morphological Heterogeneity in a Polymerization Catalyst at Multiple Reaction Stages. *Chem Catalysis* **2021**, *1*, 1413–1426.

(3) Werny, M. J.; Müller, D.; Hendriksen, C.; Chan, R.; Friederichs, N. H.; Fella, C.; Meirer, F.; Weckhuysen, B. M. Elucidating the Sectioning Fragmentation Mechanism in Silica-Supported Olefin Polymerization Catalysts with Laboratory-Based X-Ray and Electron Microscopy. *ChemCatChem* **2022**, e202200067.

(4) Smith, D. R.; Loewenstein, E. V. Optical Constants of Far Infrared Materials 3: Plastics. *Appl. Opt.* **1975**, *14*, 1335.

(5) Lin, Y.; Bilotti, E.; Bastiaansen, C. W. M.; Peijs, T. Transparent Semi-Crystalline Polymeric Materials and Their Nanocomposites: A Review. *Polym. Eng. Sci.* **2020**, *60*, 2351–2376.

(6) Rodriguez-Gonzalez, F. J.; Ramsay, B. A.; Favis, B. D. High Performance LDPE/Thermoplastic Starch Blends: A Sustainable Alternative to Pure Polyethylene. *Polymer* **2003**, *44*, 1517–1526.

(7) Guo, Y.; Wang, Z. Y.; Qiu, Q.; Su, J.; Wang, Y.; Shi, S.; Yu, Z. Theoretical and Experimental Investigations on the Temperature Dependence of the Refractive Index of Amorphous Silica. *J. Non. Cryst. Solids* **2015**, *429*, 198–201.

(8) Silica, Amorphous [MAK Value Documentation, 1991] in *The MAK-Collection for Occupational Health and Safety*; John Wiley & Sons, Ltd; **2012**, 158–179.

(9) Khlebtsov, B. N.; Khanadeev, V. A.; Khlebtsov, N. G. Determination of the Size, Concentration, and Refractive Index of Silica Nanoparticles from Turbidity Spectra. *Langmuir* **2008**, *24*, 8964–8970.

(10) Holler, M.; Diaz, A.; Guizar-Sicairos, M.; Karvinen, P.; Färm, E.; Härkönen, E.; Ritala, M.; Menzel, A.; Raabe, J.; Bunk, O. X-Ray Ptychographic Computed Tomography at 16 nm Isotropic 3D Resolution. *Sci. Rep.* **2014**, *4*, 3857.

(11) Veselý, M.; Valadian, R.; Merten Lohse, L.; Toepperwien, M.; Spiers, K.; Garrevoet, J.; Vogt, E. T. C.; Salditt, T.; Weckhuysen, B. M.; Meirer, F. 3-D X-Ray Nanotomography Reveals Different Carbon Deposition Mechanisms in a Single Catalyst Particle. *ChemCatChem* **2021**, *13*, 2494–2507.

(12) De Winter, D. A. M.; Meirer, F.; Weckhuysen, B. M. FIB-SEM Tomography Probes the Mesoscale Pore Space of an Individual Catalytic Cracking Particle. *ACS Catal.* **2016**, *6*, 3158–3167.
